# Sensory processing patterns and their relationships to coping and occupational engagement in mental health service users

**DOI:** 10.1111/1440-1630.13016

**Published:** 2025-01-21

**Authors:** Klara Forsberg, Daniel Sutton, Sigrid Stjernswärd, Catana Brown, Ulrika Bejerholm, Elisabeth Argentzell

**Affiliations:** ^1^ Department of Health Sciences, Mental Health, Activity and Participation (MAP) Lund University Lund Sweden; ^2^ Department of Psychiatry Habilitation and Aids, Region Skåne Lund Sweden; ^3^ School of Clinical Sciences Auckland University of Technology Auckland New Zealand; ^4^ Department of Occupational Therapy Midwestern University Glendale Glendale California USA

**Keywords:** mental illness, occupational therapy, sensory modulation, sensory profiles

## Abstract

**Introduction:**

Mental health service users often have sensory processing difficulties hampering their ability to cope with mental health problems and occupational engagement. However, there is little knowledge of sensory processing and its relation to these factors. Hence, this current study aims to investigate sensory processing patterns in relation to coping and occupational engagement for the target group.

**Methods:**

This cross‐sectional study involved 97 mental health service users. Sociodemographic information and self‐rated questionnaires were administered; Adolescent/Adult Sensory Profile (AASP), Coping Orientation to Problems Experienced Inventory scale (short version) (Brief‐COPE), and Profiles of Occupational Engagement among people with Severe mental illness (POES). Statistical analysis included descriptive statistics and multiple linear regression models.

**Consumer and Community Involvement:**

This study sits within an RCT project where parts were designed collaboratively with research‐educated service users.

**Results:**

The result showed strong relationships between sensory processing patterns and occupational engagement. Low levels of occupational engagement were related to high levels of *sensory sensitivity*, *sensation avoiding*, and *low registration*. Whereas, high levels of occupational engagement were related to high levels of *sensation‐seeking*. Concerning coping styles, high levels of emotion‐focused coping were related to high levels of *low registration*, while high levels of avoidant coping styles were related to high levels of *sensation‐seeking*.

**Conclusion:**

The findings indicate that occupational engagement and coping styles are related to outcomes of the sensory profile and thus affect how a person with severe mental health problems interprets and reacts to sensory stimuli in daily life.

## PLAIN LANGUAGE SUMMARY

People with mental health problems often experience difficulties in dealing with sensory input, which can reduce people's ability to do everyday activities. This study investigated how this target group experiences and responds to sensory input, and if this is related to how they cope and how active they are in daily life. The results of the study suggest that the way sensory input is experienced is related to levels of engagement in daily activities and what kinds of strategies are used to handle stressful situations. This adds to our understanding of what affects people's sensory experiences and how they respond to everyday sensations. This study indicates that the relationship between actively engaging in daily life and effectively coping with stressful situations can affect the experience and management of sensory input. The insights from the study may be used in mental health settings. As the study is small, more research is needed to confirm these conclusions.

**Key Points for Occupational Therapy**

- The level of occupational engagement is related to sensory processing patterns for people with severe mental health problems.
- Emotion‐focused coping styles are related to higher levels of *low registration* and avoidant coping styles are related to higher levels of *sensation‐seeking*.
- Coping and occupational factors should be considered when designing sensory modulation programs.

## 1 INTRODUCTION

Sensory processing is crucial for all humans as it enables the experience of internal states (interoception) and the external world (exteroception) and thus is fundamental to social and occupational engagement in everyday life (Bailliard et al., 2018; Dunn, 2001). However, people living with severe mental health problems often have sensory processing difficulties (Bailliard & Whigham, 2017; Brown et al., 2020). Qualitative research shows that these sensory processing issues hamper the ability to cope with mental health problems but also hinder engagement in everyday life and the recovery process (Andersson et al., 2021). For example, research shows that people living with mental illness often experience difficulties coping with anxiety, which is associated with hypersensitivity to certain stimuli, such as visual or auditory input (Engel‐Yeger et al., 2016). Most sensory processing occurs subconsciously, and many people with anxiety are unaware of their triggers and unable to regulate their strong autonomic responses related to specific sensory input (Bar‐Haim et al., 2007; Domschke et al., 2010). This lack of conscious awareness and the sense of being overwhelmed lead to avoidance of specific contexts, such as shopping malls, public transport, and other sites of everyday occupational engagement (Andersson et al., 2021). Although qualitative studies show relationships between sensory processing issues and aspects of coping and occupational engagement, there are currently few quantitative studies with mental health service users investigating sensory processing patterns and their relation to coping (Engel‐Yeger et al., 2016). To our knowledge, there are no quantitative studies investigating occupational engagement in relation to sensory processing.

To explain sensory processing issues, the occupational therapist, Winnie Dunn, developed a sensory processing framework (Dunn, 1997, 2001, 2014). It includes neuroscientific theories combined with behavioural and occupation‐focused theories and concepts. This framework of sensory processing is typically presented as a matrix based on two continua. The first continuum represents an individual's neurological threshold to sensory stimuli from low to high. The second continuum represents behavioural responses to sensory stimuli from a passive response to active forms of self‐regulation. The intersection of neurological threshold with the type of behavioural response creates four quadrants within the matrix, each depicting a sensory processing pattern, including: ‘low registration’, ‘sensation‐seeking’, ‘sensory sensitivity’, and ‘sensation avoiding’ (Dunn, 2014). Dunn's framework (Dunn, 2014) and the related instrument the Adolescent/Adult Sensory Profile (AASP) (Brown et al., 2001) are commonly used when studying aspects of sensory processing in people with mental health problems in occupational therapy research (Brown et al., 2020) as well as psychology research (van den Boogert et al., 2022).

A growing body of research indicates that people with mental illness have more difficulties interpreting and processing sensory stimuli than those who are mentally well (Brown et al., 2020; van den Boogert et al., 2022). A retrospective review of studies focused on comparing sensory processing patterns in people with and without mental health problems (Brown et al., 2020). The review showed that people with mental health problems had sensory processing patterns with higher levels in ‘sensory avoiding’, ‘low registration’, ‘sensory sensitivity’, and lower or equal levels in ‘sensation‐seeking’ compared to people without mental health difficulties (Brown et al., 2020). These patterns were seen across a range of diagnostic groups, including post‐traumatic stress disorder (PTSD), schizophrenia, depression, and bipolar disorder. These results (Brown et al., 2020) align with a recent meta‐analysis of sensory processing difficulties, which included autism and other neuropsychiatric conditions and showed large effect sizes in the difference between sensory processing patterns of people with and without mental illness (van den Boogert et al., 2022). Furthermore, a neuroscientific review (Harricharan et al., 2021) showed that the pattern of sensory processing in brain images appeared different in people diagnosed with PTSD compared to people without PTSD, specifically in areas important for higher cognitive functioning and emotional regulation, which potentially affect occupational engagement (Harricharan et al., 2021). While the relationship between sensory processing and mental health problems has been studied (Brown et al., 2020; van den Boogert et al., 2022), studies of sensory processing in relation to coping (Engel‐Yeger et al., 2016) and occupational engagement in mental health service users are scarce.

The concept of coping was initially developed by Lazarus and Folkman (Lazarus, 1966). Carver et al. (1989) built on the initial concept and suggested that coping strategies can be understood as the cognitive and behavioural responses a person uses when exposed to stressful situations (Carver et al., 1989). In the context of occupational therapy in mental health services and sensory processing, recent qualitative research (Andersson et al., 2021; Forsberg et al., 2024) showed that having effective coping strategies to deal with sensory issues was experienced as an enabler to engage in occupations. In another study involving 267 people living with bipolar or unipolar mood disorder, the authors used the COPE scale (Carver et al., 1989) and the AASP (Brown et al., 2001) and found weak and moderate correlations between sensory processing and coping strategies. More specifically, problem‐focused and emotion‐focused coping strategies were correlated to higher levels in the pattern of ‘sensation‐seeking’ in the unipolar group (Engel‐Yeger et al., 2016). However, the limited number of studies including coping and sensory processing in mental health service users (Engel‐Yeger et al., 2016) indicates that further research is warranted in this area.

Research within occupational therapy has shown that occupational engagement is crucial for experiencing quality of life and wellbeing when living with a mental illness (Bejerholm & Eklund, 2007; Sutton et al., 2012). A longitudinal study with 226 people based on cluster‐RCT data evaluating the occupational therapy intervention ‘Balancing everyday life’ also showed that occupational engagement, measured by the Profiles of Occupational Engagement among people with Severe mental illness (POES) (Bejerholm et al., 2006; Bejerholm & Eklund, 2006; Bejerholm & Lundgren‐Nilsson, 2015) was a strong mediator in the personal recovery process of mental health service users (Argentzell et al., 2020). Occupational engagement is defined as a multifaceted process related to involvement in daily occupations in a way that is meaningful for the individual. The meaning and level of engagement are influenced by the dynamic interplay between personal, environmental (physical and social), and occupational domains involved in occupational performance (Bejerholm & Lundgren‐Nilsson, 2015). In this sense, sensory processing patterns of an individual may affect the personal domain and thus also influence occupational engagement. Previous research indicates that sensory processing issues create occupational difficulties for mental health service users, such as problems with social participation and task attention (Bailliard & Whigham, 2017). One quasi‐experimental comparative study involving 95 people with mental illness showed that having high levels of ‘sensory sensitivity’ and ‘low registration’ were associated with lower levels of both community participation and personal recovery (Pfeiffer et al., 2014). Associations have also been found between work performance and difficulties with sensory processing for people living with ADHD (Grinblat & Rosenblum, 2022). An experiential study illustrated how people with severe mental illness experience occupational issues due to challenges with regulating and adapting to sensory stimuli (Andersson et al., 2021). This included challenges in filtering out sensory stimuli to avoid becoming overwhelmed or missing important information, which also affected occupational engagement. A further experiential study found experiences of sensory stimuli to be closely related to the capacity for occupational engagement in people living with schizophrenia (Bailliard et al., 2023). A recent study involving interviews with mental health service users who participated in a sensory modulation (SM) intervention also highlighted a relationship between sensory processing and occupational engagement (Forsberg et al., 2024). The intervention focused on supporting participants to regulate and adapt to sensory input through sensory strategies, and following the intervention participants reported both increased embodied awareness and an ability to plan engagement in daily life, based on their sensory preferences.

In summary, research in the field of sensory processing for people living with severe mental health problems is lacking, and particularly in relation to important aspects of occupational therapy practice, including coping and the ability to engage in occupations. This current study aims to fill this research gap. Therefore, the main objective of this study is to investigate sensory processing patterns and their relationships to coping and occupational engagement in mental health service users. More specifically, we aim to answer the following questions:

- Are different coping styles, including *problem‐focused coping*, *emotion‐focused coping*, and *avoidant coping*, related to sensory processing patterns?
- Are levels of *occupational engagement* related to sensory processing patterns?

## 2 METHODS

### 2.1 Ethical considerations

The study was approved by the local Research Ethics Committee, with Dnr number; 2017/43. The service users in the study received oral information and a written consent form from the course leader of the group‐based SM intervention to sign prior to participating in the larger RCT project. Participation in the study was confidential and voluntary, and the service users were informed that they could withdraw from the study without explanation at any time.

### 2.2 Positionality statement

KF is an occupational therapist and a PhD student at Lund University with a clinical employment in mental health services (MHS). DS is an occupational therapist and has a position as associate professor at Auckland University of Technology. He has experience in research related to mental health and occupational engagement, and SM. SS is a psychiatric nurse and has a position as an associate professor at Lund University. Her research focus is within the field of mental health, mindfulness, and trauma. CB is an occupational therapist and has a position as a professor emerita at Midwestern University Glendale. She has a research focus in sensory processing, mental health, and occupational therapy and has developed the instrument AASP[removed for peer review]. UB is an occupational therapist and a professor in Mental Health and Mental Health Services (MHS) and has a joint position at Lund University and MHS in Region Skåne. She is also the founder of the instrument POES. EA is an occupational therapist and has a position as an associate professor at Lund University and also has an employment in MHS. She has experience of conducting research on interventions within mental health care focusing on personal recovery, SM, and occupational balance. No authors have a relation to the participants in the study.

### 2.3 Study design

The current study is a cross‐sectional study using baseline data from a larger randomised controlled trial (RCT) evaluating a group‐based SM intervention within Swedish mental health outpatient units.

### 2.4 Participants and recruitment

The 97 mental health service users were recruited from 16 SM intervention groups in 14 mental health outpatient units, based in Southern Sweden. The inclusion criteria were being an adult (>18 years) and having severe mental health problems, which both impeded community functioning and were present for the last 2 years, according to the two‐dimensional definition of mental illness developed by Ruggeri et al. (2000). Additionally, the service users had clinically assessed moderate to severe anxiety and at least one self‐reported psychiatric diagnosis. The exclusion criteria were, not understanding or being able to communicate in the Swedish language, having treatment primarily for substance use disorder, having an intellectual disability or dementia, or being somatically or mentally acutely ill. The inclusion and exclusion criteria were assessed by the recruiting staff in mental health services, who had detailed information about the service users' health conditions and mental health status. This was done in connection with the recruitment and enrolment of participants into the larger RCT project.

### 2.5 Procedure

The service users within the larger RCT project completed the sociodemographic questionnaire and the standardised questionnaires before they started the intervention. The AASP (Brown et al., 2001) was part of the intervention, and so only the service users in the intervention group completed the instrument and were included in the current study.

### 2.6 Data collection

Data collection was mainly conducted digitally through the secured data system REDCap (Harris et al., 2009). However, a minority of the service users preferred to use pen and paper when responding to the questionnaires. The data included sociodemographic information and the standardised questionnaires; Brief‐COPE (Carver, 1997; Muhonen & Torkelson, 2013) and POES (Bejerholm et al., 2006; Bejerholm & Eklund, 2006; Bejerholm & Lundgren‐Nilsson, 2015). The data collection of the patterns of sensory processing, through the AASP (Brown et al., 2001) was done on paper, together with the occupational therapist who was the course leader in the group‐based SM intervention.

### 2.7 Questionnaires

#### 2.7.1 Sensory processing patterns

##### The AASP

The AASP (Brown et al., 2001) is an instrument with 60 items, measuring sensory processing patterns and related self‐regulation sensory strategies. The AASP is theoretically developed and based on Dunn's Sensory Processing framework (Dunn, 2014). The 60 items are divided into categories according to the sensory system (taste/smell, vestibular, proprioceptive, visual, touch, activity level, auditory) (Brown et al., 2001). Each item is related to one of the four quadrants (low registration, sensation‐seeking, sensory sensitivity, sensation avoiding), and according to Dunn's framework (Dunn, 2014) reflects variations in neurological threshold and behavioural response to sensory input. Within the four quadrants, a person can range from low (much less than most people) to high (much more than most people). For example, individuals with a high level of the ‘low registration’ quadrant need a higher amount or intensity of sensory stimuli to notice or react to input but are passive in their response and do not seek out extra input. People with high levels in ‘sensory sensitivity’ notice sensory stimuli easily and react to input that others do not notice but respond passively and become overwhelmed (Dunn, 2014).

For each item in the AASP, the participants rate to what extent they react and respond to sensory input in everyday life. This rate is done according to a 5‐point scale; 1, *almost never*; 2, *seldom*; 3, *occasionally*; 4, *frequently*; 5, *almost always*. In this current study, we calculated the sum score of each of the four quadrants (low registration, sensation‐seeking, sensory sensitivity, sensation avoiding), and each quadrant was used as a dependent variable. The maximum score in each quadrant is 75, and the minimum score is 15.

Construct validity, reliability and internal consistency were demonstrated (Brown et al., 2001). Cronbach's α in the present sample was 0.70 for low registration, 0.66 for sensation‐seeking, 0.79 for sensory sensitivity, and 0.74 for sensation avoiding.

#### 2.7.2 Coping strategies

##### Coping orientation to problems experienced inventory (Brief‐COPE)

The Brief‐COPE is an instrument reflecting individuals' coping strategies when exposed to a stressful situation (Carver, 1997; Carver et al., 1989). Brief‐COPE is the short version and consists of 14 subscales (active coping, planning, positive reframing, acceptance, humour, religion, using emotional support, using instrumental support, self‐distraction, denial, venting, substance use, behavioural disengagement, self‐blame) with two items in each scale, a total of 28 items (Carver, 1997). In each item, the participants rate to what degree they have used the coping style explained in the item on a 4‐point scale: 1, *I have not been doing this at all*; 2, *A little bit*; 3, *A medium amount*; 4, *I have been doing this a lot*. In the current study, we used the three‐factor model of the instrument where the 14 subscales are divided into three factors (Dias et al., 2012; Solberg et al., 2021): ‘Problem‐focused coping’, ‘Emotion‐focused coping’, and ‘Avoidant coping’. Sum scores were calculated by summing the person's rating in the items included in each of three factors (problem‐focused, emotion‐focused, avoidant), and each factor was used as one independent variable. The Problem‐focused coping style includes active coping strategies and includes the subscales; ‘active coping’, ‘use of instrumental support’, ‘positive reframing’, and ‘planning’. The maximum sum score in this factor is 32, and the minimum score is 8. The emotion‐focused coping style includes coping strategies aiming to regulate rather than change emotions occurring in stressful situations. The scales included in emotion‐focused coping are ‘use of emotional support’, ‘venting’, ‘humour’, ‘acceptance’, ‘self‐blame’, and ‘religion’. The maximum sum score in this factor is 48, and the minimum score is 12. The third factor, avoidant coping, includes coping strategies focusing on disengaging actively from the stressful situation. The scales included in avoidant coping are ‘self‐distraction’, ‘denial’, ‘substance use’, and ‘behavioural disengagement’. The maximum sum score in this factor is 32, and the minimum score is 8. In this current study, we used the translated [removed for peer review] version of Brief‐Cope (Muhonen & Torkelson, 2013). Reliability and internal consistency were demonstrated (Muhonen & Torkelson, 2013). Cronbach's α in the present sample was 0.75.

#### 2.7.3 Occupational engagement

##### The POES

The POES is an instrument that helps to discern the level of occupational engagement (Bejerholm et al., 2006; Bejerholm & Eklund, 2006; Bejerholm & Lundgren‐Nilsson, 2015). POES has been shown to relate to quality of life and wellbeing, mastery, locus of control, empowerment, sense of coherence, and mental health symptoms in previous cross‐sectional studies (Bejerholm & Björkman, 2010; Bejerholm & Eklund, 2007). The POES has two parts, one being the 24‐h yesterday time‐use diary, where individuals recall what they did (occupational domain), where, and with whom they performed occupations (environmental domain), and how they experienced them (personal domain). The second part consists of a self‐rated questionnaire with nine items representing different components of occupational engagement (daily rhythm of activity and rest, routines, and initiating performance, variety and range of occupations, place, social environment, social interplay, extent of meaningful occupations interpretation). Based on the time diary the participants reflect on the content and rate to what extent it corresponds to a 4‐point agreement scale; from 1, *strongly disagree* to 4, *strongly agree*, reflecting a low to a high level of engagement (Bejerholm & Lundgren‐Nilsson, 2015). In the current study, sum scores were calculated by summing the person's rating in each item, the maximum score in the POES is 36, and the minimum score is 9. The sum score was used as one independent variable. The POES has demonstrated internal construct validity and reliability. The POES self‐reported version was used in this current study (Bejerholm et al., 2006; Bejerholm & Eklund, 2006; Bejerholm & Lundgren‐Nilsson, 2015). Cronbach's α in the present sample was 0.85.

### 2.8 Data analysis

All data were downloaded from REDCap (Harris et al., 2009) and analysed using IBM's Statistical Package for the Social Sciences (SPSS) version 28. Descriptive statistics were performed, including means, median, range, and standard deviations for continuous sum scores of variables and frequencies for the categorical variable (gender). The distribution for all continuous variables was checked by histograms and showed an acceptable normal distribution. Spearman's test of correlation between the continuous sum scores of variables was completed. Four multiple linear regression models on complete cases were performed, one for each dependent variable (*low registration, sensation‐seeking, sensory sensitivity, sensation avoiding*). Since the group of non‐binary people were not included in the primary analysis because of the small size of this group (three people), sensitivity analyses were made to check if there was any difference when including them in the regression models. The assumptions of the regressions were checked for, and multicollinearity between independent variables was tested using the variance inflation factor (VIF). The residuals within the regression models were checked for autocorrelation using the Durbin–Watson test. The residuals were also checked for normal distribution, outliers, and homoscedasticity using residual plots. The significance level used in this study was *P* < 0.05 two‐tailed (Field, 2018).

## 3 RESULTS

### 3.1 Demographic characteristics of participants

The sample's demographic characteristics showed that the majority were women and had general psychiatric diagnoses (81%). Several participants had multiple coexisting diagnoses. The largest diagnostic groups were depression and anxiety disorders, followed by neuropsychiatric disorders (ADHD/ADD), PTSD, bipolar disorder, autism spectrum disorder, and personality disorders. About a fifth of the sample (19%) had been diagnosed with psychosis diagnosis. Experiences of trauma were represented in a large proportion of the sample (87%), though only 21% of the sample had a PTSD diagnosis. See Table 1 for details.

**TABLE 1:** Demographic characteristics of participants.

Characteristics	*n* (%)
Gender: Women/men/non‐binary (*n* = 97)	71/23/3 (73/24/3)
Age: Mean (*n* = 96)	44
Education (*n* = 97)	
Not completed primary school	0 (0)
Completed primary school	11 (11)
Completed high school	48 (50)
Completed university	38 (39)
Born in Sweden ‐ Yes/no (*n* = 97)	88/9 (91/9)
Present working/work‐training/studying—Yes/no (*n* = 97)	40/57 (41/59)
Self‐reported main psychiatric diagnosis (*n* = 97)	
Psychosis	18 (19)
General psychiatric diagnoses	82 (81)
Experience of trauma—Yes/no (*n* = 97)	84/13 (87/13)
Experience of repeated trauma—Yes/no (*n* = 81)	66/15 (82/18)
Type of trauma experience (*n* = 97)	
Life threatening illness or accident—Yes/no (*n* = 97)	23/74 (24/76)
Present when someone else has been killed or injured—Yes/no (*n* = 97)	21/76 (22/78)
Death of a close relative—Yes/no (*n* = 97)	45/52 (46/54)
Physical or psychological violence—Yes/no (*n* = 97)	56/41 (58/42)

### 3.2 Descriptive statistics and correlations between variables

The descriptive statistics showed that the sample in the current study had higher means in the sensory processing patterns of *low registration*, *sensory sensitivity*, and *sensation avoiding* compared to the norms (Brown et al., 2001). The mean in the pattern of *sensation‐seeking* was lower than the normative population (Brown et al., 2001). Details of descriptive statistics can be seen in Table 2, and further details of the ranges within the sensory processing patterns can be seen in Table 3.

**TABLE 2:** Descriptive statistics.

Variable	*n*	Mean (SD)	Median (min/max)
Low registration	97	39.57 (8.54)	40 (21/59)
Sensation‐seeking	97	39.95 (8.46)	39 (19/59)
Sensory sensitivity	97	48.97 (10.61)	50 (24/70)
Sensation avoiding	97	48.22 (10.26)	50 (24/68)
Age	96	44.27 (13.48)	41 (22/78)
Problem‐focused coping	93	21.09 (4.66)	21 (10/31)
Emotion‐focused coping	89	29.20 (5.86)	29 (15/43)
Avoidant coping	90	15.58 (3.16)	15 (8/25)
Occupational engagement	91	22.40 (5.64)	22 (9/36)

**TABLE 3:** Ranges within sensory processing patterns.

	Much less than most people (%)	Less than most people (%)	Similar to most people (%)	More than most people (%)	Much more than most people (%)
Sensory processing pattern					
Age 18–65 (*n* = 88)					
*Low registration*	0	1	27	31	41
*Sensation‐seeking*	11	41	41	7	0
*Sensory sensitivity*	0	3	21	23	53
*Sensation avoiding*	0	1	20	28	51
Sensory processing pattern					
Age 65 and older (*n* = 8)					
*Low registration*	0	0	37	50	13
*Sensation‐seeking*	0	25	62	13	0
*Sensory sensitivity*	0	0	38	25	38
*Sensation avoiding*	0	0	62	25	13

The directions of correlations among the mental health variables showed several statistically significant correlations between the dependent and independent variables (Table 4). The strongest correlations were between occupational engagement in a positive direction to *sensation‐seeking* and a negative direction to *sensation avoiding*. The weakest of correlations was positively directed, between *sensation‐seeking* and age. Among the sensory processing patterns (dependent variables), the strongest correlations were in a positive direction between *low registration, sensory sensitivity*, and *sensation avoiding*. See Table 4 for details of correlations.

**TABLE 4:** Correlations.

	Low registration	Sensation‐seeking	Sensory sensitivity	Sensation avoiding	Age	Problem‐focused coping	Emotion‐focused coping	Avoidant coping
Low registration								
Sensation‐seeking	0.038							
Sensory sensitivity	**0.531** ***	−0.035						
Sensation avoiding	**0.378** ***	**−**0.247*	**0.649** ***					
Age	0.070	0.019	−0.064	−0.119				
Problem‐focused coping	0.046	0.300**	0.142	0.060	−0.096			
Emotion‐focused coping	0.223*	0.259*	0.323**	0.168	−0.281**	**0.562** ***		
Avoidant coping	0.135	0.272**	0.230*	0.121	−0.154	−0.140	0.180	
Occupational engagement	−0.263**	**0.362** ***	−0.328**	**−0.425** ***	0.013	**0.391** ***	0.108	−0.205

* *P* ≤ 0.05.

** *P* ≤ 0.01.

*** *P* ≤ 0.001.

### 3.3 Multiple linear regression analysis

Four multiple linear regression models were performed, one for each of the sensory processing patterns, which were deemed as dependent variables. See details in Table 4.

The first model with *low registration* as a dependent variable showed that approximately 25% (*R* ^2^ = 0.248) of the variance in *low registration* could be explained by the model. High levels of emotion‐focused coping (*P* = 0.043) and low levels of occupational engagement (*P* = 0.004) were related to high levels of *low registration*. Women also tended to have higher levels of *low registration* than men (*P* = 0.040).

The second model with *sensation‐seeking* as a dependent variable showed that 33% (*R* ^2^ = 0.327) of the variance in *sensation‐seeking* could be explained by the model. High levels of avoidant coping (*P* < 0.001) and high levels of occupational engagement (*P* < 0.001) were related to high levels of *sensation‐seeking*.

The third model with *sensory sensitivity* as the dependent variable showed that 32% (*R* ^2^ = 0.323) of the variance in *sensory sensitivity* could be explained by the model. Low levels of occupational engagement (*P* < 0.001) were related to high levels of *sensory sensitivity*.

The fourth model with *sensation avoiding* as the dependent variable showed that 30% (*R* ^2^ = 0.298) of the variance in *sensation avoiding* could be explained by the model. Low levels of occupational engagement (*P* < 0.001) were related to high levels of *sensation avoiding*.

The assumptions for all four models were fulfilled, with normally distributed residuals, the Durbin–Watson scores were close to 2.0, which indicates no autocorrelation problems within the residuals. Furthermore, the highest VIF score was 2.025, indicating no multicollinearity problems between the independent variables in any of the four models. We applied Bonferroni correction in regression models to adjust for potential Type 1 error according to multiple comparisons (*P* = 0.013). The sensitivity analysis resulted in the same conclusion as the primary analysis and any differences were negligible.

See Table 5 for details of assumptions scores in all models.

**TABLE 5:** Multiple linear regression Models (1–4).

Dependent variables	Independent variables	β	β 95% CI	*P*
Model 1	Gender (reference men)	**4.589**	**0.222–8.957**	**0.040** *
Low registration	Age	0.091	−0.045–0.226	0.188
R: 0.498	Problem‐focused coping	−0.048	−0.576–0.480	0.856
*R* ^2^: 0.248	Emotion‐focused coping	**0.417**	**0.013–0.821**	**0.043** *
	Avoidant coping	0.023	−0.582–0.627	0.940
	Occupational engagement	**−0.504**	**−0.844–0.165**	**0.004** **
Model 2	Gender (reference men)	1.030	−3.056–5.117	0.617
Sensation‐seeking	Age	0.093	−0.034–0.220	0.151
R: 0.572	Problem‐focused coping	0.281	−0.213–0.774	0.261
*R* ^2^: 0.327	Emotion‐focused coping	0.133	−0.245–0.511	0.486
	Avoidant coping	**1.041**	**0.476–1.607**	**<0.001** ***
	Occupational engagement	**0.572**	**0.255–0.890**	**<0.001** ***
Model 3	Gender (reference men)	1.028	−4.126–6.182	0.692
Sensory sensitivity	Age	0.037	−0.123–0.198	0.643
R: 0.568	Problem‐focused coping	0.423	−0.200–1.045	0.180
*R* ^2^: 0.323	Emotion‐focused coping	0.460	−0.017–0.937	0.058
	Avoidant coping	0.391	−0.322–1.105	0.278
	Occupational engagement	**−0.829**	−**1.230–** **0.428**	**<0.001** ***
Model 4	Gender (reference men)	0.538	−4.531–5.606	0.833
Sensation avoiding	Age	−0.091	−0.249–0.066	0.251
R: 0.545	Problem‐focused coping	0.545	−0.067–1.158	0.080
*R* ^2^: 0.298	Emotion‐focused coping	0.132	−0.337–0.601	0.577
	Avoidant coping	0.059	−0.642–0.761	0.867
	Occupational engagement	**−0.938**	−**1.332–** **0.544**	**<0.001** ***

* *P* ≤ 0.05.

** *P* ≤ 0.01.

*** *P* ≤ 0.001.

## 4 DISCUSSION

This study contributes to the evidence base on the relationship between sensory processing patterns, coping styles, and occupational engagement. The results indicate that participant coping styles and levels of occupational engagement may influence their sensory processing patterns. This contrasts with earlier research, where sensory processing patterns appeared to influence participant coping styles and occupational engagement (Bailliard & Whigham, 2017; Jerome & Liss, 2005), therefore highlighting a potential bidirectional relationship between sensory‐processing, coping, and engagement in daily life.

Regarding the result of relationships between specific sensory processing patterns and coping styles, it was, for example, shown that, an increased level of emotion‐focused coping was related to an increased level of *low registration*. Emotion‐focused coping styles involve regulating feelings rather than changing stressful situations, including relatively passive strategies, such as ‘acceptance’ and ‘use of emotional support’ (Dias et al., 2012; Poulus et al., 2020). *Low registration* patterns also involve passive responses in the regulation of sensory stimuli, so this common element of passivity may be a potential reason for the relationship. The result may also indicate that people with a high level of *low registration* have such sensory difficulties that they require substantial support from others (Brown et al., 2002).

The relationship between higher levels of avoidant coping and increased levels of *sensation‐seeking* is a novel finding, as this type of relationship has not previously been reported. Past research has generally found a relationship between higher levels of sensation‐seeking and positive health‐related outcomes (Engel‐Yeger & Dunn, 2011; Jerome & Liss, 2005). Whereas avoidant coping is generally associated with negative outcomes and includes strategies that are considered maladaptive, such as ‘substance use’, ‘behavioural disengagement’, and ‘denial’ (Ambrus et al., 2020; Dias et al., 2012). One potential reason for this could be that s*ensation‐seeking* and avoidant coping involve active rather than passive behavioural strategies (e.g., substance use) to manage arousal levels and avoid or distract from stressful situations (Brown et al., 2001; Dias et al., 2012). Further, actively seeking out distracting or numbing stimuli to manage emotional overwhelm may be understandable if individuals also experience high levels of low registration, sensitivity, and/or avoidance behaviours in relation to different sensory modalities. Higher scores in the quadrants of ‘low registration’, ‘sensory sensitivity’, and ‘sensation avoiding’ are also common among people with mental health issues (Brown et al., 2020) and this aligns with the sample scores of this current study. This may explain the difference in results from previous studies into coping styles and sensory processing, which were conducted with healthy adults (Engel‐Yeger & Dunn, 2011) and university students (Jerome & Liss, 2005), rather than people with mental health problems.

In addition to the associations to coping styles, a distinct direction of results was found between levels of occupational engagement and sensory processing patterns. In relation to *sensation‐seeking*, a higher level of occupational engagement helped to explain 33% of the variance in the second regression model, while a lower level of occupational engagement affected the variance in the other three models. Low levels of occupational engagement were related to high levels of *low registration*, *sensory sensitivity*, and *sensory avoiding*. These connections between occupational engagement and the sensory processing patterns resemble previous research on sensory patterns in relation to participation and everyday life activities (Bailliard & Whigham, 2017; Pfeiffer et al., 2014). Findings from a further study also align with the results of this current study by showing relationships between high levels of ‘low registration’, ‘sensory sensitivity’, and ‘sensory avoiding’ and low adaptive functioning in daily life (Neufeld et al., 2021). Earlier research has also shown relationships between specific sensory processing patterns and difficulties in engaging in everyday life, for example, one study showed that people with severe mental health problems who have a high level of ‘low registration’ often have anxiety‐related difficulties disrupting their occupational engagement (Engel‐Yeger et al., 2016). Further, research on people with affective disorders showed similar patterns as the current study, with ‘low registration’ and ‘sensory sensitivity’ being related to greater mental health issues such as increased hopelessness and depression (Engel‐Yeger et al., 2018). An additional study (Serafini et al., 2017) found a higher level of depression related to high levels of ‘low registration’, ‘sensory sensitivity’, and ‘sensation avoiding’, while ‘sensation‐seeking’ was not related to any symptoms of depression and hopelessness, rather it seems to be protective for developing such symptoms (Engel‐Yeger et al., 2016, 2018).

The underlying concept of occupational engagement in POES may also help explain the directions in the results of the current study. In previous cross‐sectional research (Bejerholm, 2010), a higher level of occupational engagement was associated with persons with severe mental illness who had ‘just right’ opportunities for activity and was able to process activity and environmental stimuli, in alignment with the pattern of *sensory seeking*. Whereas, having a lower level of engagement was connected to few opportunities for activities and deficient processing of activity and environmental stimuli (Bejerholm, 2010), which is more aligned with the other sensory processing patterns. This dynamic nature of occupational engagement and the influence of occupational opportunities and environments are reflected in earlier experiential research (Bailliard et al., 2023) and may apply to the regression results in this current study.

No previous quantitative research has investigated sensory processing patterns in relation to occupational engagement (Andersson et al., 2021; Bailliard & Whigham, 2017), and only a few studies have explored sensory processing and coping styles (Engel‐Yeger et al., 2016; Jerome & Liss, 2005). Therefore, the current study provides new insights by showing a relationship between occupational engagement and sensory processing patterns. This finding, along with the relationship between sensory processing and certain coping strategies, indicates that sensory processing patterns might not be constant, since they are related to what types of occupation a person performs, how much, and in what social and physical environments they are engaged in. However, changes in sensory processing patterns are likely to occur over time, and further longitudinal research is needed to draw such conclusions. The findings also indicate that mental health service users may benefit from SM interventions, which target the regulation of sensory processing in the context of occupational engagement. The Sensory Awareness Program (SAP) (Forsberg et al., 2024) is one such group‐based intervention, as it focuses on sensory and occupational dynamics, as well as on supporting the recovery process in daily life for people living with severe mental health problems.

## 5 STUDY LIMITATIONS

Limitations of this study were that the sample was relatively small and included self‐reported diagnosis. These factors potentially affected the validity of the statistical results; hence, larger longitudinal studies and more diagnosis specific studies are warranted to confirm the results of the current study. There was a lack of gender diversity in the sample, with a large overrepresentation of women (73%) and much fewer men (24%). Further, the group of non‐binary people was only 3%, which did not make it possible to include them in the primary regression models. The sensory processing patterns were measured through a self‐reported questionnaire (Brown et al., 2001), which is the most common way to measure these to date. If more objective measures of sensory processing responses (i.e., physiological and behavioural) were used, it could potentially affect the results (Elwin et al., 2013).

## 6 CONCLUSIONS AND IMPLICATIONS FOR PRACTICE

This current study contributed new knowledge about the connection between sensory processing, coping styles, and occupational engagement. The results showed a relationship between low levels of occupational engagement and high levels of *low registration, sensory sensitivity*, and *sensation avoiding*, while high levels of occupational engagement were related to high levels of *sensation‐seeking*. Further, high levels of emotion‐focused coping styles were related to high levels of *low registration*, and women tended to have higher levels of *low registration* than men. High levels of avoidant coping styles were related to *sensation‐seeking*. Overall, the current study shows promising results, which imply that sensory processing patterns have a strong relationship to occupational engagement as well as certain coping strategies. This knowledge may be of value to occupational therapists working clinically in mental health services when supporting people with sensory processing issues who have difficulties with occupational engagement or coping with stressful events in daily life. An understanding of the relationships between these factors can inform intervention planning and support an occupation‐centred focus in the management of sensory processing issues. However, the result should be interpreted with caution, and further studies, including longitudinal studies, are needed to build the evidence base related to occupational engagement, coping, and sensory processing.

## AUTHOR CONTRIBUTIONS

All authors contributed to this study significantly. The last author EA designed the study and the first author KF wrote the first draft of the manuscript. The working process with analysis, interpretations, and writing up the final version of the manuscript was done collaboratively between all authors.

## CONFLICT OF INTEREST STATEMENT

The authors report no conflict of interest.

## Data Availability

The data that support the findings of this study are available on request from the corresponding author. The data are not publicly available due to privacy or ethical restrictions.
